# Oral Cancer Development in Patients with Leukoplakia – Clinicopathological Factors Affecting Outcome

**DOI:** 10.1371/journal.pone.0034773

**Published:** 2012-04-13

**Authors:** Wei Liu, Lin-Jun Shi, Lan Wu, Jin-Qiu Feng, Xi Yang, Jiang Li, Zeng-Tong Zhou, Chen-Ping Zhang

**Affiliations:** 1 Shanghai Key Laboratory of Stomatology, Department of Oral Maxillofacial–Head and Neck Oncology, Ninth People's Hospital, Shanghai Jiao Tong University School of Medicine, Shanghai, China; 2 Department of Oral Mucosal Diseases, Ninth People's Hospital, Shanghai Jiao Tong University School of Medicine, Shanghai, China; 3 Department of Preventive Dentistry, Shanghai Municipal Hospital for Oral Health, Shanghai, China; 4 Department of Oral Pathology, Ninth People's Hospital, Shanghai Jiao Tong University School of Medicine, Shanghai, China; Ludwig-Maximilians University, Germany

## Abstract

**Background:**

Oral leukoplakia (OL) is the best-known potentially malignant disorder. The objective of the current study was to evaluate the clinicopathological factors predictive of outcome in a large cohort of patients with OL, and report our experience in the early detection of malignant events.

**Methods:**

A total of 320 patients with biopsy-proven OL were retrospectively reviewed from the study institution who had a mean follow-up of 5.1 years. Data on patient and lesion at initial diagnosis and patient underwent sequential biopsies were reviewed. Multiple biopsies indicates > = 3 times sequential biopsies. Oral cancer-free survival rate (OCFS) was determined by the Kaplan-Meier method and significant factors were identified by Cox regression analysis.

**Results:**

The 3-year and 5-year OCFS was 86.6% and 82.0%, respectively. A new binary system of grading oral dysplasia was performed and Kaplan-Meier analysis indicated that high-grade dysplasia had significantly higher malignant incidence than low-grade dysplasia (5-year OCFS, 90.5% vs 59.0%; *P*<0.001), especially during the first 2–3 years of follow-up. Multivariate analysis revealed that the 4 factors including patient aged >60 years, lesion located at lateral/ventral tongue, non-homogenous lesion, high-grade dysplasia were independent significant indicators for OL malignant transformation. In addition, significant positive correlation between the multiple biopsies and these 4 factors and malignant outcome was established.

**Conclusions:**

Elderly patients with OL located at lateral/ventral tongue and who had non-homogenous lesion with high-grade dysplasia correlated much higher risk of transformation. This high-risk subpopulation was suggested to undergo sequential biopsies and histologic examination contributing to early detection of malignant event.

## Introduction

Oral cancer is the sixth most frequent leading cause of cancer death worldwide; the early detection of malignant events is a high priority for reducing deaths due to oral cancer. Oral squamous cell carcinoma (OSCC) is the most frequent form (>90%) of oral cancer, with 5-year survival rate of ∼50% despite various management in the past 3 decades [1]–[3]. It is widely accepted that development of OSCC in potentially malignant lesions evolve through a multistep process followed by varying grades of epithelial dysplasia and invasive carcinoma [4]–[6]. Thus, biopsy is supported as the standard for detecting dysplasia and carcinoma in oral lesions, and histologic assessment of oral dysplasia is currently the gold standard for determining the risk of malignant transformation. But whether the grade of oral dysplasia is significantly associated with malignant transformation remains a considerable source of debate [6]–[13]. Therefore, further studies are warranted to improve of histologic assessment of dysplasia.

Oral leukoplakia (OL) is defined as “A white plaque of questionable risk having excluded (other) known diseases or disorders that carry no increased risk for cancer" [14]. It is the most common oral potentially malignant lesion, with a higher tendency of malignant transformation increased with follow-up years [15]. Unfortunately, the risk of OL transformation is difficult to assess. Napier and Speight [16] recently reviewed clinical risk factors of OL transformation, e.g. gender, age, lesion type and location, but the results from different study populations vary. The role of tobacco and alcohol use as carcinogens related to OL malignant transformation remains controversial [17]–[20]. Hence, assessment of these potential risk factors for oral cancer development in patients with OL is still needed.

The literature focusing on longitudinal observational study of a comprehensive analysis of the clinicopathological factors predictive of outcome in patients with OL is not robust [19]–[21], whereas large cohort of patients are required to produce meaningful data supported by robust statistical analysis. Therefore the objective of the current study was to determine the clinicopathological factors predictive of outcome in a large cohort of patients with OL who received long-term follow-up (mean, 5.1 years; range, 1–20 years), and to report our experience in the early detection of malignant events in a hospital-based study of an ethnic Chinese population.

## Methods

### Patient Population

All 320 patients with biopsy-proven OL in this study were retrospectively reviewed among the archived files at the Departments of Oral Mucosal Diseases and Oral Pathology, Shanghai Ninth People's Hospital, Shanghai Jiao Tong University School of Medicine (SJTU-SM) from 1990 to 2010. All clinical information and follow-up data were obtained from the files. In our clinic, periodic follow-up examinations at intervals of every 6 months or fewer were recommended for patients with OL. Data on gender, age, lesion site, lesion type, history of smoking and alcohol intake at the initial diagnosis of OL was all reviewed. Clinically, OL were subdivided in a homogeneous type (flat, thin, and uniform) and a non-homogeneous type (verrucous, speckled, or nodular). For all the subjects, the treatments were grouped into medication (vitamin A/Chinese herb, n = 261) and surgery (n = 59). According to the World Health Organization (WHO) definition of OL [14], the exclusion criteria were as follows: (i) Any patient with a diagnosis of OL concomitant OSCC at the first visit. (ii) Any patient without the initial histologic examination of OL and the diagnosis of development of OSCC during the follow-up on site distantly located from the site of the initial biopsy; (iii) Any patient with a follow-up period of less than 12 months after initially being diagnosed with OL. (iv) Any patient with the clinical apparence and histologic features of oral white or predominantly white benign diseases, e.g. leukoedema, linea alba, leukokeratosis; and other potentially malignant diseases, e.g. lichen planus, discoid lupus erythematosus. All of the patients gave written informed consent in accordance with institutional guidelines and this study was approved by the institutional review board of Ninth People's Hospital, SJTU-SM.

### Histologic examination

All the study participants underwent biopsy. The biopsy was fixed in formalin, embedded in paraffin, and processed for routine histologic examination. The histologic examination of all subjects was determined according to the WHO criteria [22] by oral pathologist from the Department of Oral Pathology, Ninth People's Hospital, SJTU-SM. According to the binary system of grading oral dysplasia newly proposed by the WHO [7], reexamination of all the cases confirmed the diagnosis and grade of oral epithelial dysplasia. The cytology (a total of 9) and architecture (a total of 7) criteria for oral dysplasia were as previously described in literature [23]. We reclassified all cases as low-grade dysplasia and high-grade dysplasia in the current study. Classification of a lesion as low-grade was based on the observation of fewer than 5 cytological changes or fewer than 4 architectural changes. Classification of a lesion as high-grade was based on the observation of at least 5 cytological changes and 4 architectural changes.

### Statistical Analysis

In the current study, malignant transformation versus nontransformation of OL was considered to be the surrogate for the clinical outcome of the patients. Follow-up period was defined as the interval from the time when patient underwent first biopsy to the nontransformation of last consultation (for censored observations) or to malignant transformation (for uncensored observations). If the subject had any worsen changes in clinical features noted during the follow-up, sequential biopsy was performed and the biopsy with the grade of dysplasia was taken for coding. The subjects who underwent 1 time biopsy, 2 times biopsies, and multiple (> = 3) times biopsies were divided into corresponding 3 groups. Difference between groups and factors was compared by using Kruskal-Wallis test and their relationship was determined by using Pearson correlation analysis. Oral cancer-free survival (OCFS) was determined by using the Kaplan-Meier method and log-rank test. To identify risk factors in predicting risk of OL transformation, Cox proportional hazards models were utilized. Hazard ratio (HR) with 95% confidence interval (CI) and *P* value were reported. All tests were two sided, and *P* values of <0.05 were accepted for statistical significance. Statistical analyses were performed using the software packages SPSS version 16.0 for Windows (SPSS Inc., Chicago, Ill).

## Results

### Patient Characteristics

**Figure 1:**
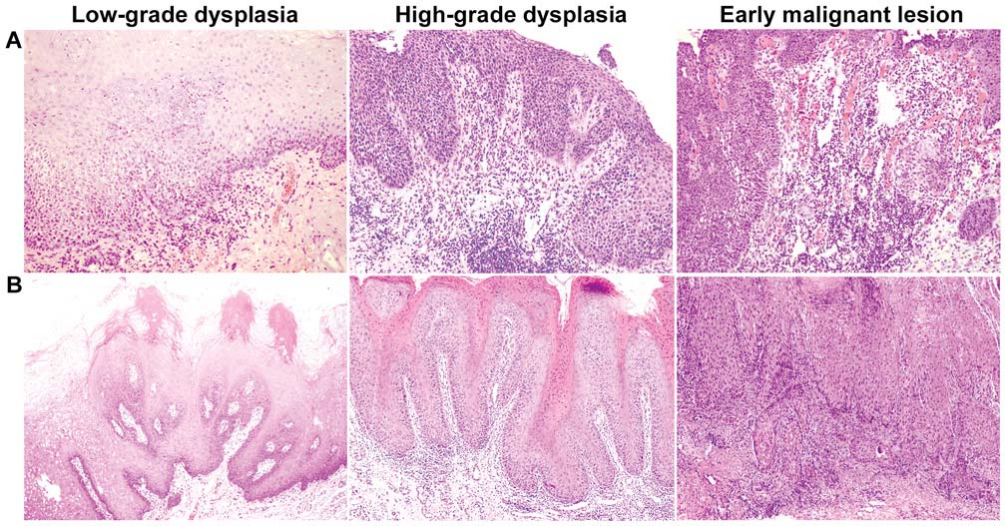
Oral cancer development in patient with leukoplakia. Malignant process of (**A**) a case of a homogenous leukoplakia and (**B**) a case of a non-homogenous verrucous leukoplakia.

A total of 320 patients with OL were identified in the current study for whom a mean follow-up of 5.1 years (range, 1–20 years) was available. Of these, 57 (17.8%) patients developed early stage I/II OSCC, with a mean interval of developing OSCC of 4.5 years. The mean interval of 27 cases of low-grade dysplasia was 5.8 years compared with that of 3.3 years of 30 cases of high-grade dysplasia (Student's t-test, *P* = 0.018). These patients were 175 females and 145 males (ratio F∶M = 1.2∶1) with the average (SD) age at initial diagnosis was 54.1 (11.6) years (range 21–83). The lateral/ventral tongue was affected in 121 (37.8%) patients, followed by the buccal mucosa (29.1%), dorsal tongue (19.4%), gingiva (8.1%), palate (3.8%). As OL were predominantly located on the lateral/ventral tongue, lesion sites were grouped as lateral/ventral tongue and others for an analysis of transformation risk. To analyze the transformation risk of the age, the cohort of patients were classified as the elderly (>60 years) and non-elderly (< = 60 years) in this study. We found that 91 (28.4%) lesions were high-grade dysplasia and only 19 (5.9%) lesions were non-homogenous type in this series (Fig. 1).

### Oral cancer-free survival analysis of risk factors in OL transformation

**Figure 2:**
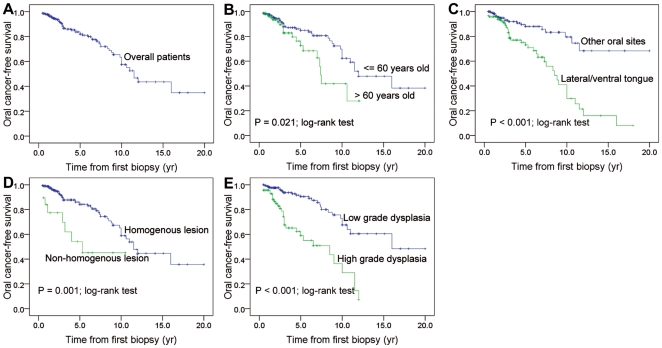
Oral cancer-free survival analysis. (**A**) by the cohort of overall patients; (**B**) by patient aged >60 years and < = 60 years; (**C**) by lateral/ventral tongue and other oral sites; (**D**) by non-homogenous and homogenous lesion; (**E**) by low-grade and high-grade dysplasia.

To investigate the time to malignant event of OL, the oral cancer-free survival (OCFS) by Kaplan-Meier method using clinicopathological factors was performed and OCFS rate at years 3, 5 were determined (Table 1). Age, lesion site, lesion type, and dysplasia were significant risk factors by log-rank test (Fig. 2). For all the 320 subjects, the 3-year and 5-year OCFS was 86.6% and 82.0%, respectively. For patient' age, elderly patient (5-year OCFS rate, 72.6%) had a higher malignant incidence than non-elderly patient (5-year OCFS rate, 84.9%) (*P* = 0.021, log-rank test). For anatomical site, it is evident that the lesion at lateral/ventral tongue (5-year OCFS rate, 73.5%) had a much higher malignant incidence than that at other sites (5-year OCFS rate, 88.0%) (*P*<0.001, log-rank test). In addition, non-homogenous OL (5-year OCFS rate, 54.2%) had a much higher malignant incidence than homogenous OL (5-year OCFS rate, 84.2%) (*P* = 0.001, log-rank test). Importantly, we observed that high-grade dysplastic lesion (5-year OCFS rate, 59.0%) was associated with an obviously increased malignant incidence compared with low-grade dysplastic lesion (5-year OCFS rate, 90.5%) (*P*<0.001, log-rank test). Of interest is that the OCFS curve showed much high incidences of malignant events for patients with high-grade dysplasia occured during the first 2–3 years of follow-up by Kaplan-Meier analysis (Fig. 2E).

**Table 1:** Oral cancer-free survival (OCFS) analysis of risk factors in transformation of oral leukoplakia.

Characteristic	Malignant events/n	OCFS rate at 3 yr (SE)	OCFS rate at 5 yr (SE)	*P*
All patients	57/320	0.866 (0.024)	0.820 (0.029)	
Gender				0.141
Female	37/175	0.865 (0.032)	0.814 (0.039)	
Male	20/145	0.867 (0.037)	0.830 (0.044)	
Age (yr)				0.021
< = 60	38/228	0.880 (0.027)	0.849 (0.031)	
>60	19/92	0.827 (0.052)	0.726 (0.071)	
Lesion site				<0.001
Other sites	19/199	0.920 (0.024)	0.880 (0.032)	
Lateral/ventral tongue	38/121	0.789 (0.046)	0.735 (0.052)	
Lesion type				0.001
Homogenous	49/301	0.877 (0.024)	0.842 (0.029)	
Non-homogenous	8/19	0.697 (0.116)	0.542 (0.132)	
Epithelial dysplasia				<0.001
Low grade	27/229	0.937 (0.020)	0.905 (0.026)	
High grade	30/91	0.675 (0.065)	0.590 (0.073)	
Smoking				0.423
Never	33/194	0.868 (0.031)	0.816 (0.039)	
Past and present	11/86	0.888 (0.044)	0.888 (0.044)	
Unknown	13/40			
Alcohol intake				0.689
Never	32/196	0.875 (0.031)	0.835 (0.037)	
Past and present	12/84	0.876 (0.045)	0.847 (0.052)	
Unknown	13/40			

SE, standard error of the mean.

### Cox regression analysis of risk factors in OL transformation

To evaluate the risk of OL malignant transformation, clinicopathological factors were analyzed by the Cox proportional hazards model (Table 2). On univariate analysis, age, lesion site, lesion type and dysplasia but not gender, smoking and alcohol intake were found to be significantly correlated with the OL transformation. To further assess the influence of each significant factor, we then did multivariate analysis and all 4 factors retained statistical significance. On multivariate analysis, elderly patient was associated with 2.17-fold (95% CI, 1.18–4.00; *P* = 0.013) increased the risk of transformation compared with non-elderly patient. The lesion located at lateral/ventral tongue was associated with 2.84-fold (95% CI, 1.55–5.18; *P* = 0.001) increased the risk of transformation compared with other oral sites. In addition, the adjusted OR for transformation was 2.55 for non-homogenous OL (95% CI, 1.15–5.68; *P* = 0.022) and 2.88 for high-grade dysplasia (95%CI, 1.64–5.07; *P*<0.001), respectively.

**Table 2:** Cox regression analysis of risk factors in transformation of oral leukoplakia.

	Univariate analysis		Multivariate analysis	
Variate	HR (95% CI)	*P*	HR (95% CI)	*P*
Gender				
Female	1.00			
Male	0.66 (0.38–1.15)	0.145		
Age (yr)				
< = 60	1.00		1.00	
>60	1.91 (1.09–3.35)	0.024	2.17 (1.18–4.00)	0.013
Lesion site				
Other sites	1.00		1.00	
Lateral/ventral tongue	3.38 (1.94–5.87)	<0.001	2.84 (1.55–5.18)	0.001
Lesion type				
Homogenous	1.00		1.00	
Non-homogenous	3.34 (1.57–7.13)	0.002	2.55 (1.15–5.68)	0.022
Epithelial dysplasia				
Low grade	1.00		1.00	
High grade	4.33 (2.55–7.36)	<0.001	2.88 (1.64–5.07)	<0.001
Smoking				
Never	1.00			
Past and present	0.76 (0.38–1.50)	0.427		
Alcohol intake				
Never	1.00			
Past and present	0.87 (0.45–1.70)	0.874		

HR, Hazard Ratio. CI: confidence interval.

### Association between multiple times biopsies and factors

To analyse the multiple times oral biopsies contributing to early detection of malignant event, association between number of biopsy times and factors and outcome was performed by using Kruskal-Wallis test and Pearson correlation analysis. The distribution of number of biopsy times of the study subjects is listed in Table 3. Significant difference in age, lesion site, lesion type, dysplasia and outcome was found (all *P*<0.05, Kruskal-Wallis test), whereas differences in gender, smoking and alcohol intake were not observed (all *P*>0.05, Kruskal-Wallis test) between the 3 groups of varying times biopsies (Table 4). Consistently, positive correlation between the 3 groups and age, lesion site, lesion type, dysplasia and outcome was significant (all *P*<0.05, Pearson's correlation test; Table 4).

**Table 3:** Number of biopsy times of the study subjects.

Oral leukoplakia	Total	UT case	MT case
All patients, n (%)	320	263 (82.2)	57 (17.8)
No. of biopsy times			
1	127	127 (48.3)	–
2	114	91 (34.6)	23 (40.4)
3	51	27 (10.3)	24 (42.1)
4	14	10 (3.8)	4 (7.0)
5	10	5 (1.9)	5 (8.8)
6	2	1 (0.4)	1 (1.8)
7	2	2 (0.8)	–

UT, untransformed case. MT, malignant transformed case.

**Table 4:** Association between multiple times biopsies and factors and outcome.

Characteristic	Total	One time biopsy	Two times biopsies	Multiple times biopsies	*P* a	*P* b
All patients, n (%)	320	127 (39.7)	114 (35.6)	79 (24.7)		
Age (yr)					0.048	0.013
<50	106	49 (46.2)	36 (34.9)	20 (18.9)		
50–59	114	47 (41.2)	36 (31.6)	31 (27.2)		
60–69	71	25 (35.2)	28 (39.4)	18 (25.4)		
> = 70	29	6 (20.7)	13 (44.8)	10 (34.5)		
Gender					0.065	0.030
Female	175	63 (36.0)	60 (34.3)	52 (29.7)		
Male	145	64 (44.1)	54 (37.2)	27 (18.6)		
Lesion site					<0.001	<0.001
Lateral/ventral tongue	121	19 (15.7)	56 (46.3)	46 (38.0)		
Other oral sites	199	108 (54.3)	58 (29.1)	33 (16.6)		
Lesion type					0.005	0.001
Homogenous	301	125 (41.5)	107 (35.5)	69 (22.9)		
Non-homogenous	19	2 (10.5)	7 (36.8)	10 (52.6)		
Epithelial dysplasia					<0.001	<0.001
Low grade	229	108 (47.2)	78 (34.1)	43 (18.8)		
High grade	91	19 (20.9)	36 (39.6)	36 (39.6)		
Smoking					0.572	0.410
Never	194	84 (43.3)	66 (34.0)	44 (22.7)		
Past and present	86	43 (50.0)	25 (29.1)	18 (20.9)		
Unknown	40					
Alcohol intake					0.367	0.161
Never	196	94 (48.0)	62 (31.6)	40 (20.4)		
Past and present	84	33 (39.3)	29 (34.5)	22 (26.2)		
Unknown	40					
Outcome					<0.001	<0.001
Cancer-free events	263	127 (48.3)	91 (34.4)	45 (17.1)		
Cancer events	57	–	23 (40.4)	34 (59.6)		

a Kruskal-Wallis test.

b Pearson correlation test.

## Discussion

To the best of our knowledge, the current study is the largest cohort of patients with OL who received long-term follow-up focusing on to determine the clinicopathological factors predictive of outcome, and this is the first to identify which subpopulation of high-risk patients with OL was suggested to undergo multiple biopsies and histologic examination in order to detect early malignant events for more aggressive management. For all the 320 subjects, the 3-year and 5-year OCFS was 86.6% and 82.0%, respectively. Multivariate analysis revealed that age, lesion site, lesion type, dysplasia were independent risk factors for OL malignant transformation. Together, elderly patients with OL located at lateral/ventral tongue and who had non-homogenous lesion with high-grade dysplasia correlated much higher risk of transformation. It highlights the importance of utilizing sequential biopsies and histologic examination to confirm the clinical diagnosis for any suspicious these high-risk lesions for early detection of malignant event.

In the current series, we considered the malignant interval elapsed from the initial diagnosis of OL to cancer development. In this context, we excluded any patient with diagnosis of OL in concomitant with oral cancer at the first visit, as well as these with a follow-up of less than 12 months after initially diagnosis with OL. A short interval from diagnosis of OL to that of cancer could lead to overestimation of the true occurrence of OL malignant transformation and potentially suggest these 2 diseases were synchronous. A frequency (17.8%) of transformation of OL into oral cancer was noted in this study, within the range reported in the literature for OL [15]. Herein, differences in treatments administered to the patients with OL were not observed, and *Cochrane Database of Systematic Review* demonstrate no evidence of effective management in preventing the transformation of OL into cancer [24]–[28].

Although the association between OL malignant transformation and clinical factors and oral habits have been accessed in the previous reports, the results from the different study populations vary. Because of our large cohort of patients with OL, it was possible to perform robust statistical analysis to identify predictors of outcome. We found that patient age is an important risk factor affecting malignant outcome, which may be correlated with genetic susceptibility contributing to the phenotype [18]. The risk of transformation was higher in the elderly patients than in the non-elderly patients in the current study. The risk of transformation of lateral/ventral tongue OL and non-homogenous OL was higher than at other sites and homogenous OL, respectively. These findings were consistent with the risk for oral location and clinical type of malignant transformation in the earlier reports [16], [29], [30].

Smoking and alcohol intake play important roles in the development of OL may be generally accepted, but the roles of these in the malignant transformation of OL remains controversial and as yet unclear. The studies by Silverman et al [19] and Schepman et al [20] reported an increased risk of transformation for the non-smoker, whereas the study by Shiu et al [17] and our current study showed that smoking was not a significant risk factor in OL transformation. Also, alcohol intake was also not a significant risk factor for OL transformation [17], [18]. Further studies are needed to investigate the potential roles of these risk factors in the malignant process of OL.

As is well-known, the histologic assessment of OL transformation is imperfect, but it has not been possible to do without it to date [7]. Actually, many clinicians currently rely on the oral epithelial dysplasia present in patients with OL as an important indicator of oral cancer risk in routine practice. Although it had been shown that patients with oral dysplastic lesions more frequent develop oral cancer than those with non-dysplastic lesions, the different grades of dysplastic OL is significantly associated with malignant transformation is conflicting [10]–[13]. At a workshop coordinated by the WHO Collaborating Centre for Oral Cancer and Precancer in the UK issues related to potentially malignant disorders of oral mucosa, a latest proposal was recommended as the two class classification (no/questionable/mild - low grade dysplasia; moderate/severe - high grade dysplasia); this view was taken that reducing the inherent subjectivity in grading oral dysplasia may enhance the likelihood of agreement between pathologists [7]. Kujan et al [23] determined the new binary system of grading dysplasia and supported this view.

In the current study, we have explored the biological significance of this binary system of grading oral dysplasia in predicting the malignant risk of OL transformation in a longitudinal large cohort, and supports the utilization of high-grade dysplasia as a significant indicator for predicting risk. This result was in agreement with that in our recent studies [31], [32]. It is noteworthy that high malignant incidences for patients with high-grade dysplasia occured during the first 2–3 years of follow-up, in similar with the findings showed by Ho et al [18] and Silverman et al [19]. This suggests that periodic follow-up during the first 2–3 years for patients with high grade dysplastic OL is important to detect early events of malignant transformation.

The another important finding in our study was that we reported our experience in the early detection of malignant events. We established significant positive correlation between the sequential multiple biopsies and age, lesion site, lesion type, dysplasia and outcome of malignant transformation, and identified a subpopulation of high-risk patients with OL was suggested to undergo multiple biopsies and histologic examination. This suggested that utilizing multiple biopsies and histologic examination to confirm the clinical diagnosis for any suspicious these high-risk lesions contribute to detect early malignant event.

In summary, the significance of the new binary system of grading oral dysplasia proposed by the WHO in the predicting risk of OL transformation was evaluated. Elderly patients with OL located at lateral/ventral tongue and who had non-homogenous lesion with high-grade dysplasia correlated much higher risk of transformation. This high-risk subpopulation was suggested to undergo multiple biopsies and histologic examination contributing to early detection of malignant event.
